# Integrin Clustering Is Driven by Mechanical Resistance from the Glycocalyx and the Substrate

**DOI:** 10.1371/journal.pcbi.1000604

**Published:** 2009-12-11

**Authors:** Matthew J. Paszek, David Boettiger, Valerie M. Weaver, Daniel A. Hammer

**Affiliations:** 1 Department of Bioengineering, University of Pennsylvania, Philadelphia, Pennsylvania, United States of America; 2 Department of Chemical and Biomolecular Engineering, University of Pennsylvania, Philadelphia, Pennsylvania, United States of America; 3 Institute for Medicine and Engineering, University of Pennsylvania, Philadelphia, Pennsylvania, United States of America; 4 Department of Microbiology, University of Pennsylvania, Philadelphia, Pennsylvania, United States of America; 5 Department of Pharmacology, University of Pennsylvania, Philadelphia, Pennsylvania, United States of America; 6 Center for Bioengineering and Tissue Regeneration, Department of Surgery, University of California, San Francisco, San Francisco, California, United States of America; 7 Department of Anatomy, University of California, San Francisco, San Francisco, California, United States of America; 8 Department of Bioengineering and Therapeutic Sciences, Institute for Regenerative Medicine and UCSF Comprehensive Cancer Center, University of California, San Francisco, San Francisco, California, United States of America; Massachusetts Institute of Technology, United States of America

## Abstract

Integrins have emerged as key sensory molecules that translate chemical and physical cues from the extracellular matrix (ECM) into biochemical signals that regulate cell behavior. Integrins function by clustering into adhesion plaques, but the molecular mechanisms that drive integrin clustering in response to interaction with the ECM remain unclear. To explore how deformations in the cell-ECM interface influence integrin clustering, we developed a spatial-temporal simulation that integrates the micro-mechanics of the cell, glycocalyx, and ECM with a simple chemical model of integrin activation and ligand interaction. Due to mechanical coupling, we find that integrin-ligand interactions are highly cooperative, and this cooperativity is sufficient to drive integrin clustering even in the absence of cytoskeletal crosslinking or homotypic integrin-integrin interactions. The glycocalyx largely mediates this cooperativity and hence may be a key regulator of integrin function. Remarkably, integrin clustering in the model is naturally responsive to the chemical and physical properties of the ECM, including ligand density, matrix rigidity, and the chemical affinity of ligand for receptor. Consistent with experimental observations, we find that integrin clustering is robust on rigid substrates with high ligand density, but is impaired on substrates that are highly compliant or have low ligand density. We thus demonstrate how integrins themselves could function as sensory molecules that begin sensing matrix properties even before large multi-molecular adhesion complexes are assembled.

## Author Summary

Critical cell decisions, including whether to live, proliferate, or assemble into tissue structures, are directed by cues from the extracellular matrix, the external protein scaffold that surrounds cells. Integrin receptors on the cell surface bind to the extracellular matrix and cluster into complexes that translate matrix cues into the set of instructions a cell follows. Using a newly developed model of the cell-matrix interface, in this work we detail a simple yet efficient mechanism by which integrins could “sense” important matrix properties, including chemical composition and mechanical stiffness, and cluster appropriately. This mechanism relies on mechanical resistance to integrin-matrix interaction provided by the glycocalyx, the slimy sugar and protein coating on the cell, as well as the stiffness of the matrix and the cell itself. In general, the resistance alters integrin-ligand reaction rates, such that integrin clustering is favored for many physiologically relevant conditions. Interestingly, the mechanical properties of the cell and ECM are altered in many prevalent diseases, such as cancer, and our work suggests how these mechanical perturbations might adversely influence integrin function.

## Introduction

Cell adhesion to the ECM is mediated by a family of heterodimeric surface receptors called integrins [1]. In addition to their function as mechanical anchors, integrins also participate in signal transduction and thereby regulate important cell behaviors, such as differentiation, motility, survival, and morphogenesis [2],[3]. To signal, integrins assemble laterally in the membrane and recruit structural and signaling proteins to form a clustered adhesion complex. In addition to their signaling function, assembled adhesion complexes also physically link the cell cytoskeleton to the ECM and transmit traction forces necessary for mechanical cell processes, such as motility and cell shape changes [4]–[7].

Both the physical and chemical properties of the ECM influence integrin adhesion complex assembly [3], [8]–[15]. The density of matrix ligands and their affinity for integrin receptors determines the number, size and distribution of integrin complexes in the cell membrane [8],[12],[15],[16]. Integrin clustering is especially sensitive to ligand spacing, as nanometer differences in the average spacing between ligands dictates whether or not integrins assemble into large adhesion complexes, such as focal adhesions [8],[16]. Matrix rigidity also regulates integrin function, as stiff matrices promote the assembly of large integrin complexes (focal adhesions) while compliant matrices support the assembly of small point-like integrin structures if any at all [13],[14]. Since integrin clustering is functionally linked to signal transduction and cell behavior, matrix-regulated adhesion assembly serves as a key sensory process that enables a cell to interrogate and respond to its extracellular environment.

Current theory holds that the adhesion complex is embedded with molecular sensors that mediate response to matrix properties. Possibilities include protein switches that undergo tension-dependent conformational changes ([17]–[20] and reviewed in [21]), as well as multivalent adaptor proteins whose incorporation into the adhesion complex are predicted to depend on factors such as matrix ligand density, matrix stiffness, and cell contractility [22]–[25].

Although receiving less attention, the integrin-ligand interaction itself could also be sensitive to the physical and chemical properties of the ECM. When both a receptor and its ligand are tethered, the kinetics and thermodynamics of complex formation depends on the intrinsic chemistry of the interaction; the distance the molecules must stretch to reach each other; and, theoretically, the compliance of the materials the molecules are tethered to [26]–[28]. This suggests that integrins could function as sensors if their aggregation is linked to bond formation with ligand.

How could integrin-ligand interaction drive integrin assembly? One popular hypothesis holds that ligand interaction induces large allosteric changes in integrins that extend to their intracellular domains (reviewed in [1],[29]). These changes in conformation could facilitate the recruitment of intracellular adaptor or signaling proteins that crosslink and cluster integrins [22],[30].

Other possibilities, however, likely exist. Following interaction with matrix-immobilized ligands, for example, integrins can assemble into complexes in a matrix-dependent manner *prior* to the recruitment of intracellular proteins [10], [31]–[33]. Consistent with this observation, a chemo-mechanical basis for how receptor-ligand interactions can drive receptor clustering independent of intracellular interactions has been described theoretically. When membranes possess two or more receptors of different lengths and chemical affinities, or possess large non-specific repellers (i.e. large proteoglycans or glycoproteins), the receptors tend to phase separate into clustered or ring-like structures upon interaction with a substrate or another membrane [34]–[37]. In essence, receptors aggregate due to a competition between receptor-mediated adhesion and non-specific repulsion that resists adhesion. Integrin clustering could therefore naturally depend on the factors that control adhesion, including ligand chemistry, matrix stiffness, and cell stiffness, and also on the factors that mediate repulsion, such as the physical properties of the glycocalyx. Hence, integrins may be able to respond to matrix properties without the necessity of auxiliary sensor proteins in the adhesion complex.

To explore if the glycocalyx can mediate integrin clustering independent of intracellular adaptors and if this clustering is responsive to the chemical and physical parameters that define the ECM, we developed a computational model of integrin-ligand interaction that includes a mechanical description of the cell-ECM interface. The model is based on the simulation algorithm called Adhesive Dynamics [38]–[45], which was originally devised to study the chemo-mechanics of receptor-mediated cell adhesion under shear flow [46]. Adhesive Dynamics models integrin-ligand bonds as Hookean springs, which allows the distance-dependent kinetic rates of bond formation and rupture to be calculated with a model developed by Bell and co-workers [26],[47]. In this work, Adhesive Dynamics was expanded to include a lattice spring model (LSM) of the cell-ECM interface. The LSM utilizes a defined lattice of nodes with interconnecting springs to calculate the elastic behavior of solid materials [48],[49]. Integration of the Adhesive Dynamics and LSM algorithms enables integrin dynamics, including force-dependent bond formation and rupture, to be explored in the context of a deformable cell-ECM interface.

Using the newly developed computational technique, we evaluate the relationship between integrin clustering, cell and glycocalyx mechanics, and the chemistry and mechanics of the matrix, and in doing so, predict that integrins themselves are responsive to matrix properties.

## Model

**Figure 1:**
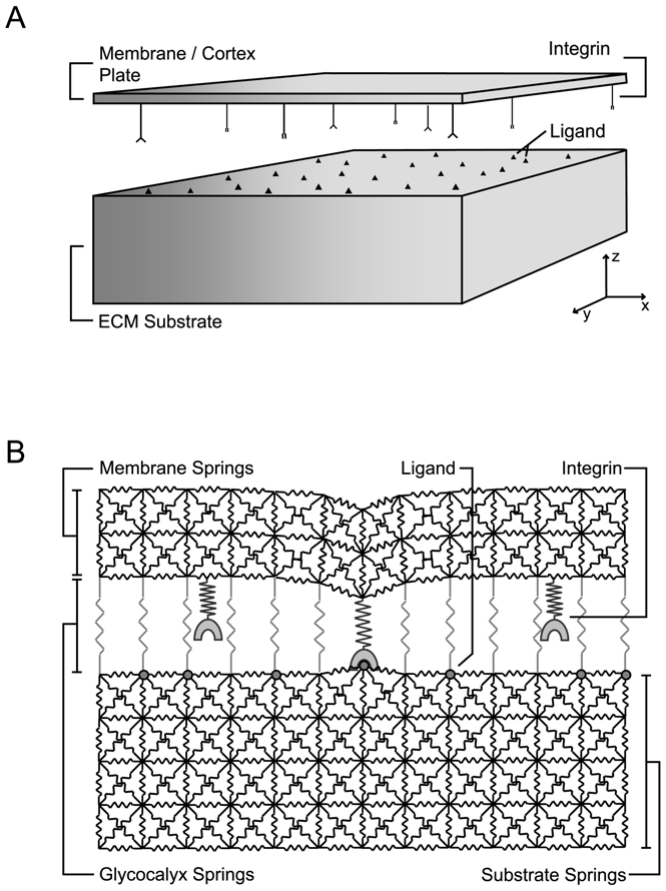
Schematics of the chemo-mechanical model of integrin dynamics. (A) Depiction of the cell-ECM interface. Mobile integrin receptors are distributed on the bottom surface of an elastic thin plate representing the cell membrane and associated actin cortex. ECM ligand sites are randomly incorporated on the top surface of an elastic substrate. Deviation from the equilibrium separation distance between the plate and substrate are resisted by a harmonic potential representing the cellular glycocalyx. During the simulation, integrin receptors switch between inactive and active conformations, and active integrins can bind ECM ligands. Free integrins not bound to the matrix can also diffuse along the cell surface. Formation of integrin-ligand bonds can induce mechanical deformations in the plate and substrate. (B) Depiction of the lattice spring model (LSM) used to numerically calculate the stress-strain behavior in the interface. Simple cubic lattices of nodes are fit to the ECM substrate and membrane/cortex plate and all nearest and next nearest nodes in each lattice are connected by springs to represent the solid mechanics of these materials. Additional springs between the nodes in the top of the substrate and bottom of the plate are added to describe the mechanics of the glycocalyx as a simple harmonic potential. Some nodes on the top surface of the substrate LSM are designated as ligand binding sites. Integrin-ligand bonds are represented by additional spring connections between these ligand sites and the bottom of the membrane/cortex LSM.

A chemo-mechanical model of integrin dynamics was developed to describe the stochastic formation and rupture of integrin bonds within a deformable cell-ECM interface. Kinetic Monte Carlo (KMC) was used to simulate integrin diffusion, changes in integrin activation status, and bonding interactions between cellular integrins and matrix ligands. In many cell types, integrin binding and clustering occurs following an initial weak adhesive interaction between the cellular glycocalyx and the ECM substrate that establishes close contact, a condition in which the outer boundary of the cell (i.e. the glycocalyx) is physically in contact with the ECM [31],[50],[51]. Consequently, we simulated integrin dynamics in a region of the cell and ECM already in close contact. The physical picture of the model is diagrammed in Figure 1A. The cell membrane and the surface of the ECM substrate were initially flat, apposed parallel to each other, and separated by a distance equivalent to the thickness of the cellular glycocalyx. Within the cell-ECM interface, integrin receptors were randomly distributed on the surface of the membrane and ECM ligands were distributed on and tethered to the substrate surface. Over the course of the simulation, integrins diffused and formed bonds with the ECM substrate.

Since the rates of adhesive bond formation and rupture will depend on the distance between the molecules and the deformability of the materials they are tethered to, a mechanical model of the cell-ECM interface was constructed. The interface was described by a flat, isotropic, elastic, solid plate that represented the bending mechanics of cell membrane and associated cortex; a thick, isotropic, elastic, flat-surfaced substrate that modeled the matrix substrate; and a repulsive potential between the plate and substrate that described the non-specific cell-matrix repulsion mediated by the glycocalyx (Figure 1A; [47], [52]–[54]). For simplicity the membrane and cortex were treated as a single mechanical entity. Complications, such as the membrane peeling away from the cortex, were not considered in the current work, although, they could be addressed in future derivations of the model. Since the intracellular cytoskeletal network is considerably softer than the cortex, it should minimally influence membrane/cortex deformations induced by small integrin-mediated adhesion forces in the absence of cell contractility [55], and thus was neglected in the model.

The glycocalyx-mediated repulsion between the cell and ECM arises from a combination of several effects, including the electrostatic repulsion of negatively charged glycocalyx polymers, osmotic pressure due to squeezing out of water molecules from the hydrated glycocalyx layer, and steric compression of glycocalyx polymer chains [47]. We took the approach of Agrawal and Radhakrishnan and lumped these effects into a single term of mechanical resistance described by the following harmonic potential per unit differential area:
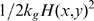
(1)Here, *H* is the local compression of the glycocalyx and *k_g_* is the effective stiffness constant per unit area that takes into account the combination of effects described [56].

As in prior Adhesive Dynamics simulations, integrin-ligand bonds were modeled as individual Hookean springs that connect the cell to the matrix substrate (Figure 1B; [40],[42],[46]). In the current model bonds were added by connecting a spring between the bottom surface of the membrane cortex plate and the top surface of the matrix substrate. The force on these bonds and the resulting deformation in the cell-ECM interface were governed by the material properties of the interface, including the bending modulus of the cell membrane/cortex plate, the stiffness of the matrix substrate, and the stiffness and equilibrium thickness of the repulsive potential representing the glycocalyx.

### Calculation of the stress-strain behavior of the cell-ECM interface model

Stress and strain in the interface were calculated with the LSM numerical method. The LSM is a computationally-efficient mesoscopic approach frequently used in fracture mechanics that utilizes a system of regularly spaced nodes and interconnecting harmonic springs to model the mechanical behavior of solids (reviewed in [49]). When the node lattice, arrangement of spring connections, and spring constants are chosen correctly, the large-scale behavior of the LSM directly maps onto linear elasticity theory [48]. The LSM is numerically equivalent to a finite element model that has simple linear elements [48]; however, we employ the LSM methodology over the more commonly used finite element method for two primary reasons. First, the integrin-ligand bonds are described by discrete springs [46], and these springs can be easily incorporated into the LSM. Second, the LSM avoids computationally expensive remeshing algorithms, which a finite element method would need to call upon each time bond formation or rupture occurred in the interface.

To implement the LSM, a node and spring model was constructed for both the membrane/cortex plate and the ECM substrate, as shown in Figure 1B. Nodes were placed on an initially cubic lattice and all nearest {1 0 0} and next-nearest {1 1 0} neighbor nodes were connected by Hookean springs, each having the same spring constant. In response to stress, springs could pivot freely and the nodes could undergo translational movements that minimized the potential energy of the spring network. A system configured in this manner behaves as an isotropic elastic solid that has a fixed Poisson's ratio *ν* = 1/4 and an adjustable Young's modulus:
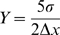
(2)where Δ*x* is the LSM lattice node spacing and *σ* is the Hookean spring constant [57],[58]. If Δ*x* is small compared to the length scale of interest, the spring system approximates an elastic continuum. The actin cortex and ECM, however, are not continuous on the protein-length scale, which is relevant to integrin-ligand interaction. To better reflect the micro-architecture of cell-ECM interface, we used an LSM lattice spacing of 20 nm, which is on the order of the size of a matrix protein or cytoskeletal filament. Changes in the lattice spacing by an order of magnitude, though, were not expected to alter the qualitative nature of our results if the spring constants were also adjusted to maintain the Young's moduli.

In all simulations unless otherwise noted, a 1.4 µm×1.4 µm area of the cell membrane was simulated. A 40-nm thick membrane/cortex plate and a 400-nm thick ECM substrate spanning this area were constructed using a 70×70×3 and a 70×70×21 node LSM, respectively (Figure 1B). The springs of each LSM were assigned a spring constant (Table 1) that achieved the desired material rigidity (Equation 2). The harmonic potential (Equation 1) between the membrane and substrate, i.e. the glycocalyx, was added to the model by incorporating additional linear springs between the ECM-substrate and membrane-spring networks. To add the springs, the plate and substrate networks were aligned and each node in the top surface of the substrate network was connected by a Hookean spring to the node directly above it in the bottom surface of the plate network (Figure 1B). The equilibrium spring length of these connections was set equal to the desired thickness of the glycocalyx. The spring constant of the connections, *σ_g_*, was related to the effective compressibility of the glycocalyx layer (See Equation 1) by the following expression:
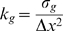
(3)


**Table 1:** Model parameters.

Parameter	Definition	Best Estimate	Reference
*σ_g_*	Glycocalyx spring constant	0.02 pN/nm	[56]
*σ_m_*	Membrane spring constant	0.4 pN/nm	[72],[73],[92]
*σ_b_*	Bond spring constant	2 pN/nm	[40],[93]
*l_g_*	Glycocalyx thickness	43 nm	[52],[53]
*l_b_*	Equilibrium bond length	27 nm	[94],[95]
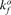	Unstressed intrinsic on-rate	1×10^5^ s^−1^	[62],[63]
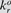	Unstressed intrinsic off-rate	0.01 s^−1^	[62],[63]
*γ*	Reactive compliance	0.4 nm	[68]
*k_a_*	Integrin activation rate	0.5 s^−1^	[30],[96]
*k_d_*	Integrin de-activation rate	5 s^−1^	[30],[96]
*D*	Integrin diffusion coefficient	5×10^4^ nm^2^/s	[97]
*k_b_T*	Thermal energy	4.28 pN·nm	
*R*	Integrin receptor density	100 #/µm^2^	[98]
*L*	Ligand density	<2500 #/µm^2^	[99]

Bonds were modeled as Hookean springs and were added to the LSM by connecting the desired node in the top surface of the matrix LSM with a node in the bottom surface of the membrane/cortex LSM. Likewise, a bond was removed by removing the appropriate spring from the model.

The deformations in the LSM caused by bond formation were calculated by relaxing the entire spring network to mechanical equilibrium. The potential energy stored in the LSM was given by
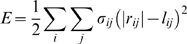
(4)where the summation *i* is over all nodes in the system, the summation *j* is over all nodes connected to node *i*, |*r_ij_*| is the distance between node *i* and *j*, and *σ_ij_* and *l_ij_* are the spring constant and equilibrium length of the spring connecting node *i* and *j*. The system energy was minimized when the vector sum of forces on each node that can undergo translation was zero, which was achieved by iteratively solving the following system of equations
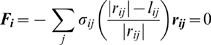
(5)


For relaxation, periodic boundary conditions were applied to the LSM nodes forming the lateral sides of the substrate and membrane/cortex networks. Under this condition, which was implemented to limit finite-size effects, the material strain induced by a stress at one side of the network propagates in a mirror-like fashion on the opposite side of the network.

### Chemical reactions and simulation of integrin dynamics

Initially, ligand binding sites and integrin receptors were distributed uniformly and randomly in the cell-ECM interface. Nodes on the top surface of the substrate LSM were selected at random and designated as ligand binding sites until the desired ligand density was achieved (Figure 1B). Since the lattice spacing was 20 nm, the maximum ligand density was 2500 #/µm^2^, which is approximately the saturating density for large ECM proteins, such as fibronectin or collagen, absorbed on flat substrates, such as tissue culture plastic or glass slides [59],[60]. Integrin receptors were placed randomly on the bottom surface of the membrane/cortex plate, but unlike the ECM ligands, the positions of free integrins (not bound to ligand) were not limited to sites of LSM nodes.

Three integrin states were described in the model that reflect the major conformational states integrins are known to adopt: “inactive” (low-affinity), “active” (high-affinity), and ligand occupied [61]. Although inactive integrins can bind soluble ligands in *in vitro* binding assays [62],[63], when locked in the inactive conformation through molecular engineering and expressed on the cell surface, integrins (α_IIb_β_3_ and α_v_β_3_) do not bind tethered ligands [64]. We thus made the assumption that only active integrins can bind ligand to reflect the relatively low probability of bond formation between matrix-tethered ligands and inactive integrins on the cell surface. Four integrin reactions were therefore modeled in our simulation: activation of inactive integrins, deactivation of active integrins, bond formation between active integrin and ligand, and bond dissociation. In addition, integrin “hop” reactions were included to describe the diffusive movements of unbound integrins.

The conversion between active and inactive integrin states was described by simple transition rates, *k_a_* and *k_i_*, which describe, respectively, the rate of conformational change from the inactive to active and active to inactive states. In the cell, the dynamic equilibrium between active and inactive integrin states depends on a variety of factors, including divalent cations, cell signaling, and intracellular integrin binding partners such as talin. *k_a_* and *k_i_* in this model can be viewed as phenomenological parameters that take all these influences into account.

The distance-dependent rates of bond formation and rupture were calculated according to the equations formulated by Bell and co-workers [26],[47]. As mentioned, integrin-ligand bonds were modeled as Hookean springs. For such a bond, the reverse reaction rate in the Bell model takes the form of:
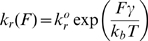
(6)where 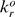 is the unstressed intrinsic dissociation rate, *F* is the force on the bond, and γ is an empirically measured quantity with units of length describing the bond's sensitivity to force [26],[28]. The bond force was calculated using Hooke's law based on the equilibrium extension of the bond as determined by Equation 5. Since the force on each bond could be unique, an individual rupture rate for each bond was calculated.

Association rates were calculated for each active integrin and ligand in close proximity. Integrin ligand binding partners that were separated by a lateral cutoff distance greater than 10 nm in the xy-plane were assigned an association rate of exactly zero. For pairs that were within the cutoff, the bond formation rate directly followed from the Boltzmann distribution for affinity [65] and was given by:
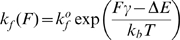
(7)where 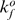 is the unstressed intrinsic association rate and Δ*E* is the minimum mechanical potential energy change resulting from bond formation [47]. To calculate *F* and Δ*E* for a specific pair of binding partners, a bond spring was temporally connected to the desired ligand site, and the system was relaxed to equilibrium. Δ*E* was then calculated according to Equation 4 and *F* was determined by Hooke's law.

Diffusion of unbound integrins (inactive and active unbound) was modeled by hop reactions in the plane of the membrane (bottom surface of LSM plate). As originally proposed by Elf and Ehrenberg [66], the hops were of discrete length Δℓ and occurred along the four directions defined by the positive and negative x- and y- axes. The rate for a specific integrin to undergo a hop reaction was given by:
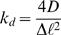
(8)where *D* is the diffusion coefficient for integrins in the membrane (10^−10^ cm^2^/s). The length Δℓ that we used in the simulation is 5 nm, which is on the order of the diameter of the integrin molecule. Periodic boundaries were employed for receptor diffusion to limit finite-size effects.

**Figure 2:**
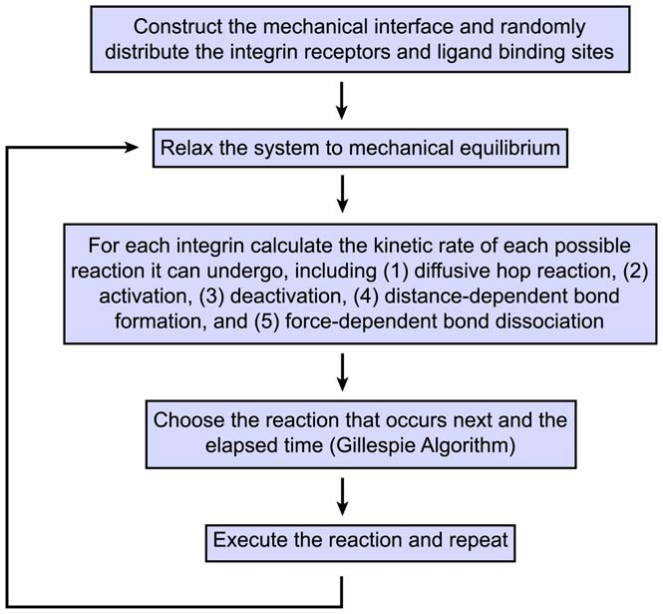
Flow-diagram of the simulation algorithm.

The time evolution of the system was simulated by kinetic Monte Carlo according to the Gillespie algorithm [67]. For a given chemical and mechanical state of the system, the Gillespie algorithm determined the reaction that occurred next and the time that elapsed until that reaction occurred. Reactions were selected through random number sampling of a probability distribution constructed based on the kinetic rates of all possible reactions. The system ultimately was evolved through an iterative process of calculating the reaction rates for the current system state, selecting the next reaction, executing the reaction, updating the rates, and repeating (Figure 2).

To determine the next reaction and the variable time step once the reaction rates were calculated, two random numbers *ran_1_* and *ran_2_* were generated from a uniform probability distribution between 0 and 1. The next reaction, μ, was selected according to:
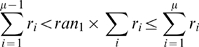
(9)where 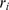 is the rate constant for a particular reaction involving a specific integrin and the summation *i* is over all possible reactions. The time that elapsed between the last reaction and the newly selected reaction was given by:
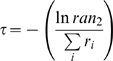
(10)After the next reaction and *τ* were determined, the selected reaction was executed. Either an integrin was moved by Δ*ℓ* in a randomly selected direction (hop reaction), the activity state of the integrin was flipped (activation or deactivation reaction), or a bond was incorporated or removed from the LSM at the appropriate ligand site (bond formation or dissociation). The simulation time was then incremented by *τ*, the spring network was relaxed back to mechanical equilibrium using Equation 5, and the reaction rates were again calculated. This procedure was repeated until the desired simulation time elapsed (Figure 2).

### Algorithm optimization and approximation

The mechanical energy minimization defined by Equation 5 was computationally expensive. Two main optimizations were thus implemented to reduce the frequency of calls to the minimization algorithm and increase its efficiency. First, energy minimums were stored to memory upon calculation to avoid repeatedly minimizing the same configuration of integrin bonds. Second, smaller sub-systems of springs and nodes were minimized, as opposed to the entire spring network. Since strain induced by an integrin-ligand bond vanished with sufficient distance from the bond, the total system potential energy minimum could be computed by minimizing a smaller sub-region surrounding the bond. The distance from a bond at which the strain vanished depended on the physical parameters defining the system, and hence sub-system size was optimized for a particular set of matrix, membrane/cortex, glycocalyx, and bond parameters. Typical sub-systems ranged from 400–800 nm in dimension. For minimization with Equation 5, nodes at the boundary of a sub-system were constrained to their current location to implement the vanishing strain boundary condition.

A small number of simulations were executed on rigid matrix substrates that had reaction interfaces spanning a membrane area greater than 1.4 µm×1.4 µm. Solutions for these larger systems were obtained by approximating the distance-dependent rate of integrin-ligand bond formation. Specifically, the minimum change in system potential energy, Δ*E*, and the equilibrium force on the integrin ligand bond, *F*, necessary to compute the bond formation rate were estimated based on the equilibrium separation distance between the unbound integrin and ligand. This approximation avoided the necessity of repeatedly minimizing the system energy to calculate bond formation rates.

In order to estimate Δ*E* and *F*, the dependence of Δ*E* and *F* on equilibrium integrin-ligand separation distance was first determined. To do so, integrin-ligand bonds were randomly and sequentially added to a model cell-ECM interface. For each integrin-ligand bond added, the equilibrium integrin-ligand separation distance before binding and the equilibrium bond force and change in system potential energy after binding were recorded. Plots of *F* versus initial separation distance and Δ*E* versus initial separation distance squared were each well-fit by quadratic equations (Figure S1), at least over the range of physical parameters utilized in this work. Because bonds were added to the system randomly, the relationships did not depend on a specific configuration of bonds. The relationships were dependent, however, on the model's physical parameters, and thus force and energy relationships were determined for each combination of physical parameters examined. During simulation of integrin dynamics, the curve-fits were used to estimate Δ*E* and *F* as a function of equilibrium integrin-ligand separation distance to calculate the bond formation rates.

For best-estimate system parameters (See below), the average errors in approximating Δ*E* and *F* based on curve fits were 6.3% and 3.5%, respectively, corresponding to an average error in bond formation rate of 3.7% according to Equation 7. Results from simulations of integrin dynamics with estimated Δ*E* and *F* were not statistically different from those in which rates were calculated by directly minimizing the system energy (Figure S2).

### Parameters

Table 1 lists the parameters that were used in the simulations unless otherwise noted. The dynamic integrin parameters were based on those reported for fibronectin and the α_5_β_1_ integrin. Other possible integrin parameters, however, were also considered to extend the relevance of the model results to other types of integrins and cell surface receptors. For α_5_β_1_, the kinetic rates of the integrin-ligand interaction, the force-dependence of the interaction, the mobility of the integrin in the membrane, and the density of integrin on the cell surface have been reported [62], [63], [68]–[70]. The rates of integrin activation and deactivation, however, have not been measured experimentally. Based on experimental reports of the equilibrium distribution of inactive and active integrins on the cell surface [30],[64], the free energy of conformational change was approximated to be 2–3 *k_b_*T [44]. Considerations of molecular diffusion rates provide an upper limit of ∼1 s for the large structural movement that occurs during activation [71]. We thus used estimates of 0.5 s^−1^ and 5 s^−1^ for the activation and deactivation rates, respectively, although other possibilities were evaluated.

The springs comprising the membrane/cortex plate were assigned a Hookean constant that achieved the experimentally measured flexural rigidity (bending modulus) of the actin cortex, 1×10^−19^ N·m [72],[73]. The Hookean constant was related to the flexural rigidity, *I*, of the plate by:
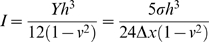
(11)where *h* is the thickness of the plate.

The Hookean constants of the ECM substrate springs were varied according to Equation 1 to achieve elastic moduli in the physiological range of 10^2^–10^5^ Pa [13],[74]. Since cellular experiments are typically conducted on extremely rigid non-deformable glass or plastic substrates, we also constructed non-deformable substrates in our model by assigning an arbitrarily large spring constant of 1000 pN/nm. This approximates a substrate with a Young's modulus of roughly 0.1 GPa (for comparison, glass or tissue culture plastic is ∼1 GPa [13]).

The thickness of the glycocalyx is reported to be approximately 40–50 nm [52] and up to 100 nm for certain cell types such as endothelial cells [53]. In this model, a best estimate of 43 nm was used for the glycocalyx spring length, i.e. its thickness, but other values were considered. While the stiffness of the glycocalyx has not yet been measured directly, estimates are available. Agrawal and Radhakrishnan estimated the glycocalyx stiffness by fitting simulations of nano-particle adhesion on the cell surface to analogous experimental data. Based on these results we estimated *σ_g_* to be 0.02 pN/nm [56]. This estimate is in good agreement with purely theoretical estimates calculated by considering the statistical mechanics of chain molecules anchored to a surface [47]. Like the glycocalyx thickness, additional possibilities for *σ_g_* were explored.

### Data analysis

To analyze the extent of integrin clustering, a two-dimensional point pattern analysis of the integrin membrane positions projected onto the xy-plane was constructed. The analysis was performed using Ripley's K-function [75],[76], which measures the extent to which a point pattern deviates from a random Poisson distribution and is given by:
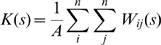
(12)where the summations *i* and *j* are over all integrin point positions, *A* is the projected area of the membrane, *s* is the sampling radius, and *W_ij_*(*s*) is exactly equal to one if the distance between points *i* and *j* is less than *s* and zero otherwise. Periodic boundaries were utilized in the calculation of *W_ij_*(*s*). To facilitate the interpretation of the statistic, these data were transformed [77] into the following form:
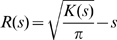
(13)For a point pattern with complete spatial randomness, *R*(*s*) has an expected value of zero, and if the points are clustered *R*(*s*) has a positive value. The maximal value of *R*(*s*) and the radius at which the function is maximal provide a measure of the degree of clustering and the cluster size respectively.

To analyze the degree of cooperativity in integrin-ligand binding interactions, Hill plots of the steady state bond fraction versus ligand density were constructed. The plots were fit to a version of the Hill equation that also accounts for the possibility of ligand depletion:
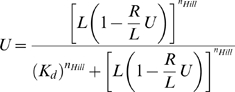
(14)where *U* is the bond fraction, *R* is the total integrin receptor density, *L* is the total ligand density, K*_d_* is the bond dissociation constant and *n_Hill_* is the Hill coefficient. The model was fit to the Hill plots for both K*_d_* and *n_Hill_* with non-linear least squares regression.

## Results

### Interface mechanics and cooperative integrin binding

**Figure 3:**
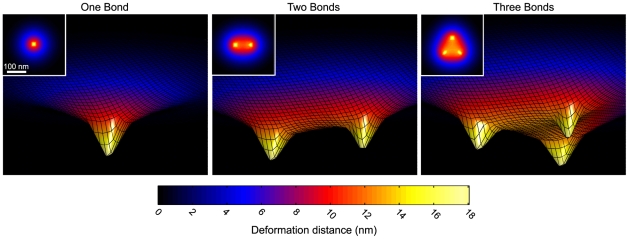
Membrane deformations in response to integrin-ligand bonds. The membrane surface is depicted in the presence of one, two, or three bonds with a rigid substrate (xy- and z- coordinates are not to scale); *σ_s_* = 1000 pN/nm, *l_g_* = 45 nm, *σ_g_* = 0.01 pN/nm. The inlays are the corresponding xy- contour maps of the z- membrane displacements. Note that larger areas of the membrane are brought in closer proximity to the ECM substrate when more bonds are placed in close proximity to each other.

Integrin-ligand binding rates are dependent on the distance the molecules must stretch to reach each other. By inducing mechanical deformations, adhesive bond formation could modify these distances and therefore be cooperative due to mechanical coupling. In order to determine how the cell membrane/cortex deforms during binding, we calculated the equilibrium deformations that were induced by the addition of integrin-ligand bonds into our mechanical model of the cell, glycocalyx, and matrix. A single integrin bond between the cell and a rigid ECM substrate caused a highly localized deformation that extended laterally in the plane of the membrane approximately 150 nm from the bound site (Figure 3). The placement of additional bonds in the deformed region pulled a significantly larger area of the cell into closer proximity with the matrix substrate (Figure 3). We thus imagined that new bond formation would be most favorable nearby existing bonds, since the distance between integrins and ligands would be reduced in this area, and that bond formation would become increasingly favorable as additional bonds accumulated together and induced larger deformations.

**Figure 4:**
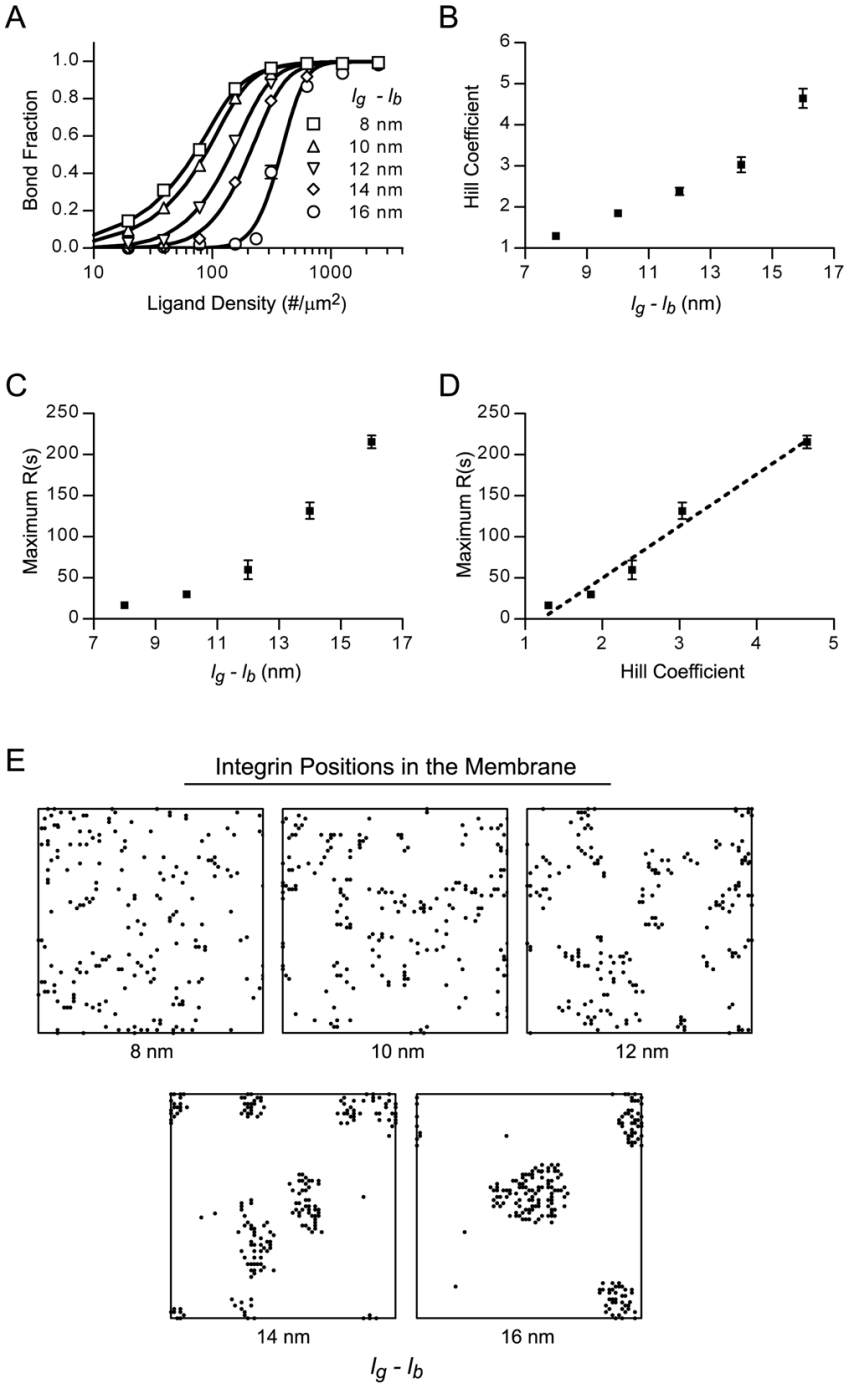
The glycocalyx mediates integrin binding cooperativity and clustering on rigid matrixes. (A) Hill plots of the steady-state integrin bond fraction versus ligand density for various effective glycocalyx thicknesses (*l_g_*−*l_b_*). The best-fit lines to the Hill equation are also shown. Bond fractions were determined by simulating integrin dynamics on rigid ECM substrates; 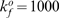 s^−1^. (B) The Hill coefficients derived from non-linear least squares curve fitting of the Hill plots. (C) The extent of integrin clustering, as indicated by the Ripley K-statistic (maximum *R(s)*), as a function of effective glycocalyx thickness. (D) Correlation between integrin binding cooperativity (n_Hill_) and the extent of integrin clustering. (E) Maps of the steady-state xy- integrin positions in the membrane for different effective glycocalyx thicknesses.

To test if bond formation was indeed cooperative, we ran simulations of integrin dynamics on rigid ECM substrates of varying ligand density and constructed Hill plots of the steady-state bond fraction (Figure 4A). Since the thickness of the glycocalyx determines the initial distance between integrin and ligand partners, the effective thickness of the glycocalyx (*l_g_−l_b_*) was also varied in an attempt to manipulate cooperativity. Hill plots were constructed from these simulation results and were fit to a form of the Hill equation which accounts for low ligand density (Equation 14). The best-fit Hill coefficients were greater than one, indicating cooperative integrin binding, and increased with enhanced glycocalyx thickness (Figure 4B).

Cooperative integrin-ligand interactions resulted in a clustered pattern of integrin bonds, as can be seen Figure 4E, which shows integrin positions after 30 minutes of simulation on rigid substrates (L - 2500 #/µm^2^). With increasing glycocalyx thickness and hence more cooperative integrin-ligand interactions, integrin clusters became fewer in number, larger in size, and more densely packed with integrins (Figure 4E). To quantify the extent of clustering, we preformed a point-pattern analysis on the steady-state integrin positions by calculating the maximum of the transformed Ripley K-function, *R(s)*. Maximum values greater than zero indicate that the integrins are clustered and the magnitude of the value is related to the degree of integrin clustering. Our point-pattern analysis demonstrated that the degree of clustering increased with enhanced glycocalyx thickness (Figure 4C) and was proportional to the level of cooperativity, as indicated by the Hill coefficient (Figure 4D).

**Figure 5:**
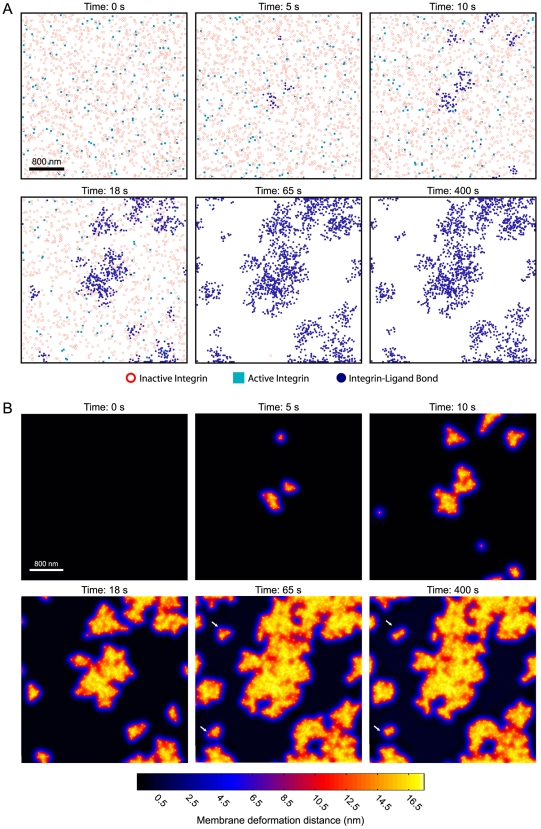
Chemical and mechanical evolution of the integrin system. The plots in (A) are temporal snapshots of the xy- positions of inactive integrins (red circles), active unbound integrins (light blue squares), and bound integrins (dark blue dots) obtained during simulation of integrin dynamics on a rigid ECM substrate with best-estimate parameters (Table 1). The corresponding equilibrium z-direction membrane deformations are depicted in (B). Simulated area: 3 µm×3 µm.

**Figure 6:**
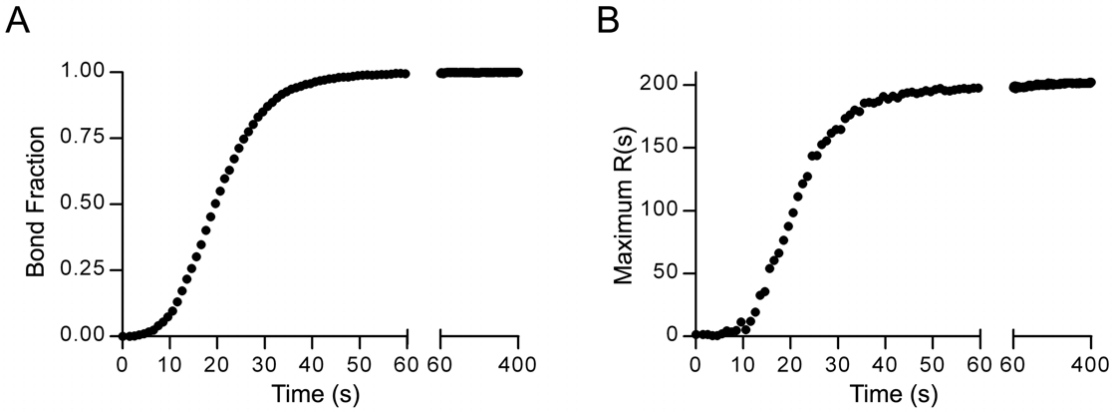
Kinetic profiles of bond formation and clustering. Plots showing the kinetic profiles of integrin bond formation (A) and the extent of integrin clustering (B) generated by simulating integrin dynamics on a rigid matrix with best-estimate parameters (Table 1).

Kinetically, integrin clusters typically formed within tens of seconds to minutes of simulated time. Figure 5 shows the chemo-mechanical evolution of the integrin system for best-estimate parameters (Table 1) on a rigid matrix (L - 2,500 #/µm^2^). As Figure 5 demonstrates, new bonds formed rapidly in regions of the cell-ECM interface deformed by prior bonds and formed slowly in regions devoid of bonds. The bonds began to form after approximately a ten second delay, at which point the rate of bond formation accelerated until saturation was reached after approximately 50 seconds (Figure 6A). The statistical measure of integrin clustering, max *R(s)*, exhibited a similar kinetic profile to that of the bond fraction, indicating that clustering was primarily driven by bond formation (Figure 6B).

While integrin clustering was primarily driven by the initial binding of integrins to the matrix, integrins continued to condense in the clusters over a much slower time-scale due to bond rearrangements occurring through repeated cycles of bond breakage and reformation (See white arrows – Figure 5B; See also the slow upward rise in Ripley statistic – Figure 6B). Hence, integrin clustering was biphasic and characterized by an initial fast bond formation and clustering step, followed by a slow bond rearrangement and condensing process.

### Interplay between integrin adhesion and glycocalyx repulsion determines integrin clustering

**Figure 7:**
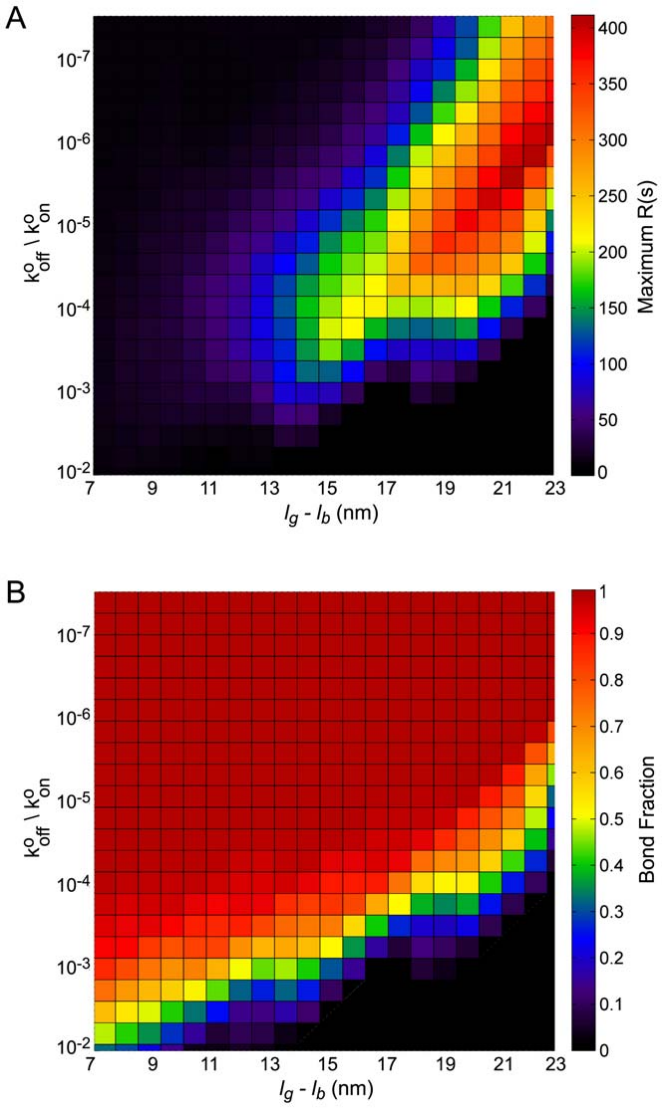
Integrin-ligand affinity, glycocalyx thickness, and bond length control integrin bond formation and clustering. The maximal Ripley clustering statistic (A) and equilibrium integrin bond fraction (B) resulting from simulations on rigid substrates with various combinations of effective glycocalyx thickness (*l_g_*−*l_b_*) and integrin-ligand affinity (See Table 1 for parameters not depicted).

Since integrin clustering required both integrin-ligand adhesion and cell-ECM repulsion, we mapped the relationship between integrin-ligand affinity and glycocalyx-mediated cell-ECM repulsion. To do so, we ran simulations in which the glycocalyx-thickness and chemical-affinity parameter space was systematically varied. As shown in Figure 7, clustering depended strongly on receptor-ligand affinity and the effective thickness of the glycocalyx. For high-affinity interactions but relatively thin glycocalyxes, the majority of integrin receptors were bound but not clustered (Compare Figure 7A and 7B). When the receptor-ligand affinity was relatively low, integrin receptors were neither bound nor clustered. If the glycocalyx was relatively thick compared to the integrin bond length and the receptor-ligand interaction was of sufficient affinity, however, integrins bound ligand and assembled into clusters. Integrin clustering was particularly sensitive to variations in glycocalyx thickness and bond length, as small changes of five to ten nanometers in the effective thickness of the glycocalyx could switch the integrin system from clustered to unclustered or vice versa.

**Figure 8:**
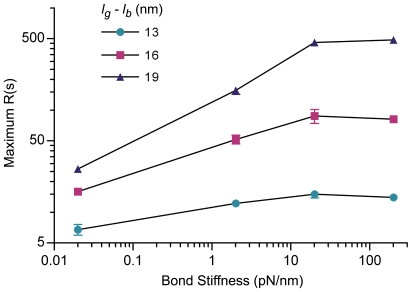
Bond length and stiffness control integrin clustering. Quantification of steady-state integrin clustering in simulations run with various values of bond stiffness (*σ_b_*) and bond length (effective glycocalyx thickness; *l_g_*−*l_b_*). All additional simulation parameters are best-estimate and listed in Table 1.

While integrins are able to switch between activity states, this property was not essential for integrin clustering. For best estimate parameters, integrins clustered if they were constitutively maintained in the active, ligand-binding conformation or instead were allowed to switch between inactive and active states (data not shown). Receptor density was modified to control the number of active receptors available for binding. Although clusters were smaller and less frequent in number for lower initial densities (Figure S4), integrins generally clustered when the initial receptor density was high or low. Increased integrin bond stiffness, however, generally enhanced clustering (Figure 8 and S3). Integrin clustering in our simulations thus was controlled by integrin bond length (Figure 7, 8, and S3), bond stiffness (Figure 8 and S3), and affinity for ligand (Figure 7), which all depend or are predicted to depend on integrin activation state. These results suggest a functional link between integrin conformation, glycocalyx properties, and integrin clustering.

For physiologically-relevant parameters, integrins clustered even when the total initial receptor density was reduced by a factor of ten (Figure S4). These results indicate that integrins could still cluster in the presence of soluble ligand, which would effectively reduce the number of available receptors to bind matrix-tethered ligands, if the soluble ligand concentration was non-saturating.

### Integrin clustering is responsive to matrix ligand density

Experimentally, small nanometer differences in average ligand-ligand spacing were shown to dictate the strength of cell adhesion and whether or not integrins cluster [8],[15],[16]. In our model, we found that the cellular deformations induced by bond formation, which are required for cooperativity, extend laterally only a limited distance (∼150 nm) from the bond in the plane of the membrane (Figure 3), and that this distance is on the order of the maximum ligand spacing reported to support integrin clustering (∼73 nm; [16]). Since integrins may not be able to utilize cooperative binding to cluster if ligands are spaced too sparsely, we sought to determine the relationship between integrin clustering and ligand spacing and how this relationship was controlled by the mechanics of the cell and glycocalyx.

**Figure 9:**
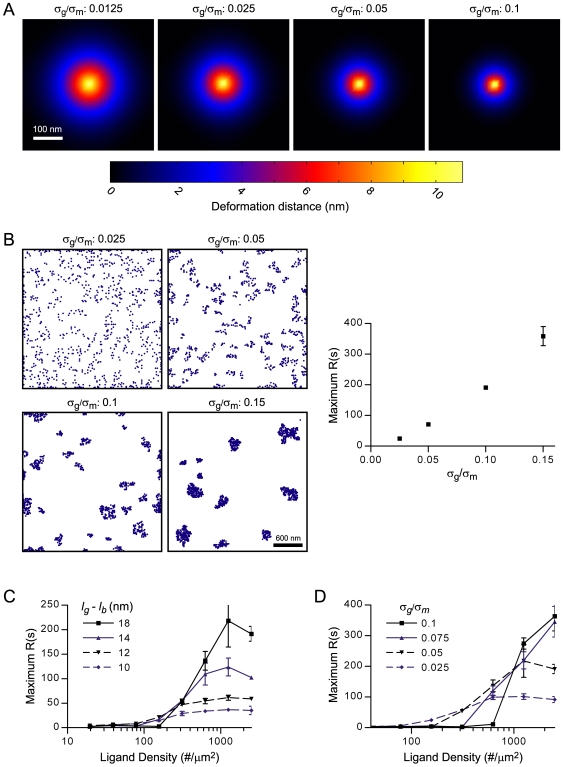
Cell and glycocalyx stiffness modulate integrin clustering and response to ligand spacing. For various values of glycocalyx stiffness, (A) depicts xy-maps of the magnitude of z- direction membrane deformations that occur in response to a single integrin bond between the membrane/cortex and a rigid ECM substrate; *l_g_* = 39 nm. (B) depicts the steady-state integrin positions for various *σ*_g_/*σ*_m_ ratios acquired by simulating integrin dynamics on a rigid ECM substrate; simulated area = 2 µm×2 µm. For various effective glycocalyx thicknesses, (C) plots the steady-state maximal Ripley clustering statistic against ligand density for integrin simulations on rigid substrates; 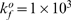 s^−1^. (D) plots the steady-state Ripley clustering statistic against ligand density for various values of glycocalyx stiffness; 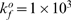 s^−1^.

We first tested how mechanical parameters, including the glycocalyx stiffness, glycocalyx thickness, and membrane/cortical rigidity, affected the lateral width of the cell deformation induced by bond formation. We observed that varying the glycocalyx thickness over a physiological range of possibilities impacted the magnitude of the z-direction height of membrane/cortex deformation above the substrate, but only had a minimal impact on the xy-width of the deformation (data not shown). The ratio of the glycocalyx stiffness (*σ_g_*) to the membrane/cortex stiffness (*σ_m_*), however, did influence the deformation width, as a decrease in *σ_g_*/*σ_m_* was associated with a larger in-plane deformation of the membrane/cortex plate (Figure 9A).

We next ran integrin simulations on rigid ECM substrates while varying either glycocalyx thickness or the glycocalyx to membrane/cortex stiffness ratio. For best-estimate *σ_g_*/*σ_m_* (Table 1), a threshold ligand density approximately of 200 #/µm^2^ was required for integrin clustering regardless of glycocalyx thickness (Figure 9C). This value corresponds to an average intermolecular ligand spacing of 71 nm. Manipulating *σ_g_*/*σ_m_*, though, altered the minimal ligand density necessary to support clustering. As suggested by the cell deformations (Figure 9A), enhancing the stiffness ratio shifted the minimal ligand density to higher values (Figure 9D). Glycocalyx stiffness also influenced the characteristics of integrin clusters. Similar to increasing glycocalyx thickness, increasing glycocalyx stiffness resulted in enhanced integrin-binding cooperativity, more extensive integrin clustering, and the formation of more tightly-packed clusters of integrin (Figure 9B and S4).

### Integrin clustering is sensitive to the stiffness of the ECM substrate

**Figure 10:**
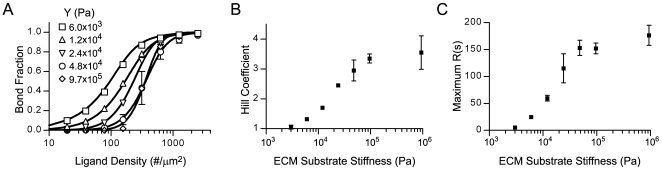
Integrin binding cooperativity and clustering are diminished on compliant ECM substrates. (A) Hill plots and the corresponding best-fit lines to the Hill equation for integrin simulations on ECM substrates of varying stiffness as indicated by the Young's modulus, Y; 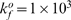 s^−1^. The corresponding best-fit Hill coefficients (B) and maximum Ripley clustering statistic (C) as a function of substrate stiffness, demonstrating that integrin binding cooperativity and clustering are sensitive to the rigidity of the ECM substrate.

To test the effect of matrix stiffness on the formation of adhesive bonds and on integrin clustering, we ran dynamic integrin simulations on ECM substrates of varying stiffness. Hill plots of the simulation results were constructed (Figure 10A) and fit to the Hill equation accounting for ligand depletion (Equation 14). We found that an ECM substrate with a Young's modulus of at least 2000 Pa was required to support cooperative integrin binding (Figure 10B). More compliant substrates failed to promote cooperative binding because the highly flexible ligands facilitated fast rates of association between integrin and ligand regardless of position in relation to other bonds. For substrates stiffer than 2000 Pa, the Hill coefficients for integrin binding increased nearly linearly with the logarithm of the substrate stiffness until reaching a plateau at approximately 100,000 Pa (Figure 10B). The extent of integrin clustering, max *R(s)*, was correlated with the observed Hill coefficients (Figure 10C), indicating that substrate rigidity controls integrin binding cooperative and clustering. These results suggest one possible mechanism of how integrins could “sense” matrix rigidity.

## Discussion

In this work, we built a new model to study integrin adhesion and clustering that couples the chemistry of bond formation with the mechanics of a composite, layered material representing the cell membrane/cortex, glycocalyx, and ECM. The biology incorporated into the model was basic and included only integrin activation/deactivation and association/dissociation reactions. Despite the simplicity of the molecular interactions, when coupled to the mechanics of the system, our model exhibited complex integrin adhesion behaviors that match those reported in the experimental literature. These behaviors can be explained by one simple principle: when deformations in the cell membrane or ECM accompany bond formation, the distance-dependent kinetic rates for other potential integrin-ligand binding interactions are modified. In essence, integrin bonds pull the cell membrane and ECM substrate into closer proximity and new bonds form more readily in these deformed regions. We showed that for realistic model parameters, clustering was sensitive to both the physical and chemical properties of the matrix, suggesting a simple yet efficient mechanism by which integrin adhesions sense matrix properties.

Integrin clustering in our model was driven by the interplay between integrin-mediated adhesion and glycocalyx-mediated cell-ECM repulsion. While a relationship between integrin-ligand affinity and integrin clustering has been suggested [10], [80]–[82], we now show that the thickness and stiffness of the glycocalyx may regulate this relationship. Indeed, we found that manipulating glycocalyx thickness/stiffness parameters while maintaining the intrinsic integrin-ligand affinity can switch the integrin system from an unclustered state to a clustered state or vice-versa. Similarly, changes in integrin-ligand affinity could also induce a switch in integrin clustering state depending on glycocalyx parameters. Furthermore, changes in integrin length, such as the structural extension that occurs during activation, could change the effective thickness of the glycocalyx to also modulate integrin clustering. In general, high-affinity integrin-ligand interactions in the context of a relatively thick or stiff glycocalyx promoted integrin clustering. A glycocalyx too thick or rigid, however, impeded bond formation and thereby prevented clustering. These results suggest the glycocalyx is a potent regulator of integrin system behavior and signaling. Such a relationship may be extremely important in diseases such as breast cancer, in which 95% of the cells have modified glycocalyx composition or structure and in which integrin clustering is functionally-linked to loss of tissue homeostasis and the development of a malignant phenotype [13],[83].

Our model provides one explanation for the exquisite sensitivity integrins exhibit in response to variations in matrix-ligand density [8],[15],[16]. In cellular experiments on rigid ligand-coated substrates, integrins cluster when the average intermolecular ligand spacing is less than or equal to 58 nm, but not when it is greater than or equal to 73 nm [16]. These results have fueled the notion that cells posses molecular “rulers” that mediate this chemo-sensory process. Our model suggests that the ruler might actually be the cell membrane and associated actin cortex rather than a specific molecule, such as an adhesion plaque protein. In order for integrins to cluster, we found that the average spacing between ligand molecules had to be less than the lateral width of the membrane/cortex deformation induced by an integrin bond. If the deformation was too small relative to the ligand spacing, integrin-ligand binding was not cooperative and integrins did not cluster. For our best-estimate mechanical parameters, the width of cell deformation induced by an integrin bond (150 nm) was on the order of the experimentally-measured ligand spacing at which the unclustered-to-clustered integrin transition occurs experimentally. Moreover, when best-estimate mechanical parameters were utilized in simulations of integrin dynamics, we found that an average intermolecular ligand spacing of 71 nm was necessary to drive integrin clustering in our model, which is in excellent agreement with experimental results. The width of the cell surface deformation was primarily determined by the ratio of the glycocalyx stiffness to membrane/cortex thickness, and hence this ratio controlled the threshold ligand density required for integrin clustering. We thus propose that the integrin adhesion system may be intrinsically sensitive to ligand density and that this sensitivity may be tuned by the mechanical properties of the cell and glycocalyx.

We also found that integrin clustering was responsive to matrix stiffness. On progressively more compliant substrates, the rate of integrin-ligand bond formation was increasingly fast due to the enhanced flexibility of the ligand binding site. Consequently bond formation was not cooperative on highly compliant substrates, since new bonds could readily form in the interface regardless of proximity to pre-existing bonds. After evaluating a range of matrix stiffnesses, we determined that integrin clustering in our model requires a substrate with a Young's modulus of at least 2000 Pa, at which point the extent of clustering increases with the logarithm of substrate stiffness until maximum clustering is achieved around 100,000 Pa. These results agree well with cellular experiments conducted on ECM-functionalized hydrogels of tunable stiffness, on which integrins assemble into larger and more numerous adhesions on matrices above 1000 Pa [13],[14],[84]. Furthermore, cell behaviors correlated with integrin clustering, such as cell spreading, demonstrate an incremental response to increases in matrix stiffness between approximately 1000 Pa to 50,000 Pa, which is again in agreement with the sensitivity range for integrin clustering predicted in this work [13],[84]. While integrin-mediated matrix mechano-sensing has been assumed to require actomyosin contractility to generate matrix probing forces and adhesion plaque proteins to respond to these force (reviewed in [21],[85]), our model would suggest that integrin themselves can respond to matrix stiffness in one manner independent of myosin or plaque proteins.

Experimentally-observed features of integrin clustering, such as its sensitivity to matrix properties, were recapitulated in our model without the incorporation of cytoskeletal adaptor proteins into the model. Indeed, for best-estimate parameters, the kinetic profiles of integrin bond formation and clustering simulated by our model recapitulate the short delay in integrin bond formation observed experimentally when the cell first contacts the ECM, as well as the fast rate of *de novo* integrin adhesion assembly and clustering observed in cells [10],[31],[50],[78],[79]. This does not suggest, however, that cytoskeletal interactions are insignificant. Many lines of experimental evidence clearly demonstrate that cytoskeletal interactions regulate the size and signaling activity of integrin adhesion structures (reviewed in [86]). We envision that integrin-cytoskeletal interactions could synergize with the mechanically-coupled integrin-ligand interactions described in this work to drive a more robust integrin clustering response with heightened sensitivity to matrix properties or with additional levels of regulation. Our model, however, does offer an explanation for how integrins can cluster prior to recruiting cytoskeletal adaptor proteins, as has been observed in time lapse studies of adhesion complex assembly [10],[31],[50]. Similar to the kinetics of integrin assembly in these time-lapse studies, integrins in our model spontaneously clustered on rigid substrates in tens of seconds to minutes even though cytoskeletal interactions were not included in the model. Provocatively, since clustering was sensitive to matrix properties, our results suggest that integrins may begin to sense matrix properties prior to the assembly of more advanced adhesion structures, such as focal complexes and focal adhesions [86].

It is well-documented that force promotes integrin adhesion complex assembly, which raises the question of whether cytoskeletal forces would influence the myosin-independent integrin clustering described in this work. In our model, integrins cluster because one bond pays a portion of the energy penalty associated with compressing the glycocalyx for the next integrin to complex a nearby ligand site. Myosin-driven forces on integrin bonds could actively pull the cell and ECM into closer spatial proximity, and hence pay this energy penalty [21]. In the context of our integrin clustering model, these force-driven deformations should enhance integrin bond formation and aggregation to possibly achieve states of integrin cluster size or density that would otherwise be unlikely. Similarly, exogenously applied forces to the cell, such as fluid shear forces in the vasculature, could induce deformations in the cell-ECM interface that modify integrin clustering response. Therefore, both endogenous contractile forces and exogenous applied forces could influence integrin distribution through a mechanism similar to that proposed in this work.

Many aspects of integrin clustering described by our model are justified experimentally. For example, reports have demonstrated that receptor-ligand interactions are distance-dependent [27],[28] and that the cell and ECM are in closest proximity at sites containing integrin adhesions [87]–[89]. Perhaps some of the best support of the model is provided by studies with biomimetic lipid vesicles. When lipid vesicles functionalized with adhesion molecules and a repulsive brush border are brought in contact with a complimentary solid surface, receptor-ligand bonds cluster despite the simple chemistry of the vesicle system [90],[91]. Since the repulsive brush border is required for patterned bond formation, these studies suggest that adhesive bond clustering results from the interplay between adhesion and repulsion, as our model predicts.

Several novel predictions stemming from our model, however, must still be validated experimentally. This includes determining if matrix rigidity controls integrin clustering by altering kinetic rates of bond formation, evaluating if cell and glycocalyx stiffness controls the relationship between integrin clustering and ligand density, and determining if the glycocalyx is indeed a potent regulator of integrin function and clustering. Testing these predictions should provide significant insight into how cell adhesions sense and respond to their ECM environment.

In conclusion, we showed how the coupling between the chemistry of bond formation and the mechanics of the cell and glycocalyx may drive integrin clustering in a matrix-dependent manner. Our results suggest a mechanism by which integrins function as sensors of matrix rigidity and chemistry.

## Supporting Information

Figure S1 Relationship between bond force, potential energy change, and unbound integrin-ligand separation distance. (A) Schematic showing the equilibrium separation distance, d, between the tip of an unbound integrin and a ligand. The relationships between d and the bond force and equilibrium change in potential energy after bond formation are depicted in (B) for several combinations of glycocalyx thickness and stiffness. To generate the plots in (B), integrin bonds were sequentially and randomly added to a 240 nm×240 nm region of a cell-ECM interface having a rigid substrate. For each bond added, the initial separation distance, d, was recorded as well as the equilibrium force on the newly formed bond and the incremental change potential energy between mechanical equilibrium states. Bond force versus initial separation distance and change in potential energy versus separation distance squared were well-fit to quadratic equations (fits shown in red), as indicated by high R2 values displayed on each plot. Physical parameters not listed are best-estimate and shown in Table 1.
(1.34 MB TIF)

Figure S2 Accuracy of simulation results using estimated bond formation rates. (A) Steady-state integrin positions in the membrane resulting from simulation of integrin dynamics in which bond formation rates (Equation 7) were calculated using curve-fits, such as those shown in Figure S1, to estimate bond force and potential energy change as a function of unbound integrin-ligand equilibrium separation distance (See Model Development - Algorithm Optimization and Approximation). (B) Steady-state integrin positions from simulations in which bond formation rates were determined by minimizing system energy using Equation 7 (See Model Development - Chemical Reactions and Simulation of Integrin Dynamics). (C) Quantification of steady-state integrin clustering in simulations with best-estimate parameters in which bond formation rates were estimated or rigorously calculated.
(0.50 MB TIF)

Figure S3 Bond length and stiffness regulate integrin clustering. Steady-state integrin positions acquired by simulating integrin dynamics on rigid substrates with various combinations of integrin bond length and stiffness. See Table 1 for additional parameters. Simulated area: 2 µm×2 µm.
(1.25 MB TIF)

Figure S4 Influence of initial receptor density on integrin clustering. Steady-state integrin positions determined by simulating integrin dynamics on rigid substrates with various initial densities of integrin receptor. All other parameters are best-estimate and listed in Table 1. Simulated area: 2 µm×2 µm.
(0.83 MB TIF)
