# Supplementary figures and images for: Integrin Clustering Is Driven by Mechanical Resistance from the Glycocalyx and the Substrate
Source: PLoS Comput Biol. 2009 Dec 11;5(12):e1000604. doi: 10.1371/journal.pcbi.1000604 (PMC2782178; doi:10.1371/journal.pcbi.1000604)

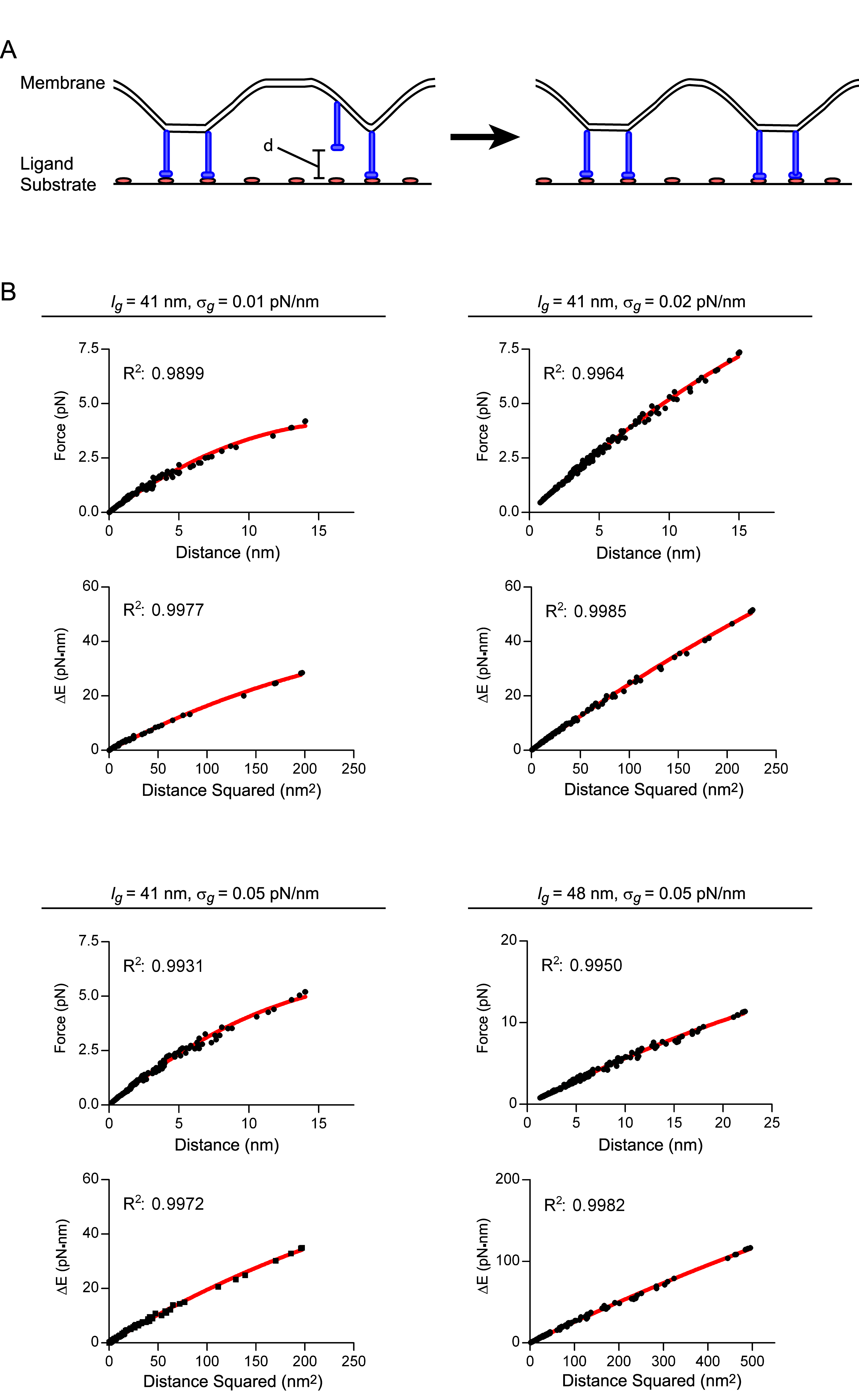

Supplement: Figure S1 — Relationship between bond force, potential energy change, and unbound integrin-ligand separation distance. (A) Schematic showing the equilibrium separation distance, d, between the tip of an unbound integrin and a ligand. The relationships between d and the bond force and equilibrium change in potential energy after bond formation are depicted in (B) for several combinations of glycocalyx thickness and stiffness. To generate the plots in (B), integrin bonds were sequentially and randomly added to a 240 nm×240 nm region of a cell-ECM interface having a rigid substrate. For each bond added, the initial separation distance, d, was recorded as well as the equilibrium force on the newly formed bond and the incremental change potential energy between mechanical equilibrium states. Bond force versus initial separation distance and change in potential energy versus separation distance squared were well-fit to quadratic equations (fits shown in red), as indicated by high R2 values displayed on each plot. Physical parameters not listed are best-estimate and shown in Table 1. (1.34 MB TIF) [file pcbi.1000604.s001.tif]

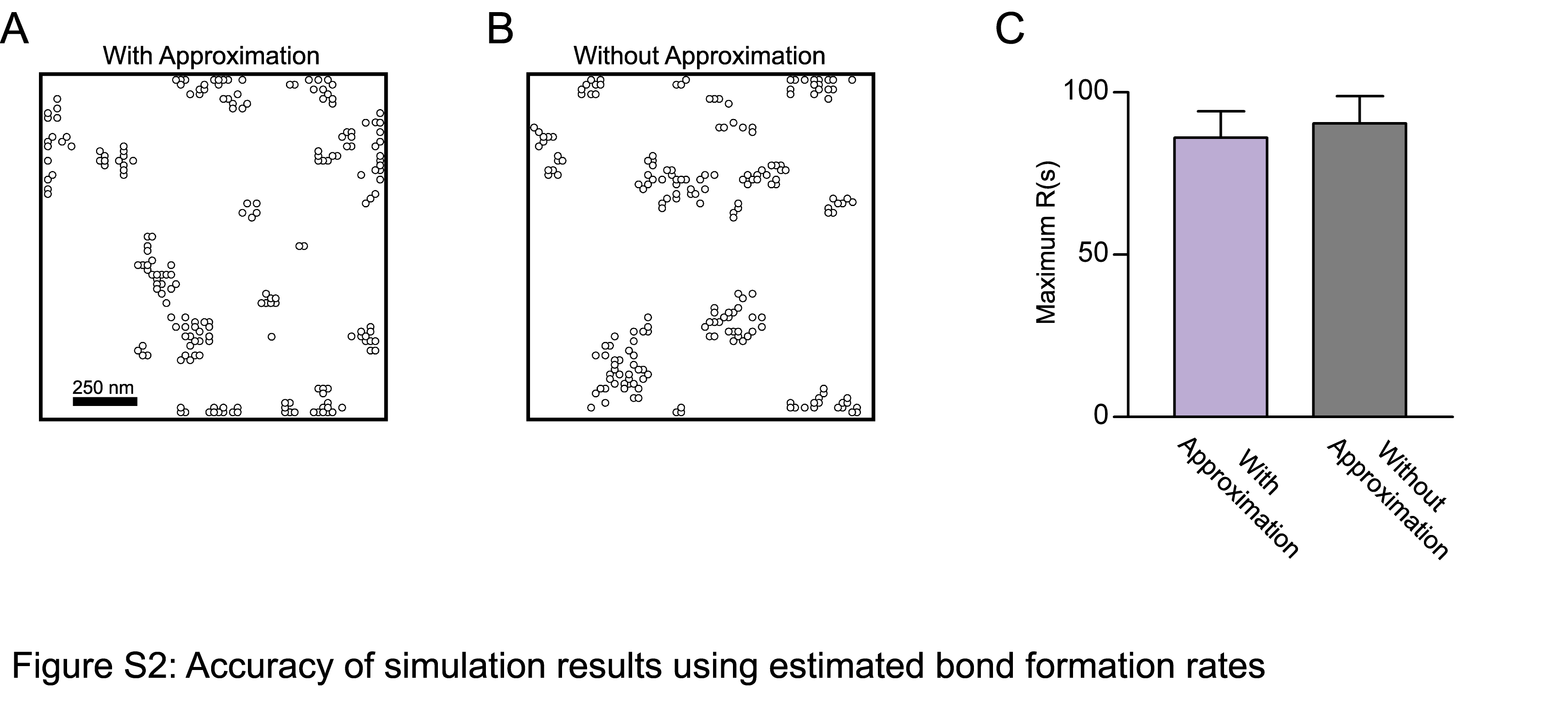

Supplement: Figure S2 — Accuracy of simulation results using estimated bond formation rates. (A) Steady-state integrin positions in the membrane resulting from simulation of integrin dynamics in which bond formation rates (Equation 7) were calculated using curve-fits, such as those shown in Figure S1, to estimate bond force and potential energy change as a function of unbound integrin-ligand equilibrium separation distance (See Model Development - Algorithm Optimization and Approximation). (B) Steady-state integrin positions from simulations in which bond formation rates were determined by minimizing system energy using Equation 7 (See Model Development - Chemical Reactions and Simulation of Integrin Dynamics). (C) Quantification of steady-state integrin clustering in simulations with best-estimate parameters in which bond formation rates were estimated or rigorously calculated. (0.50 MB TIF) [file pcbi.1000604.s002.tif]

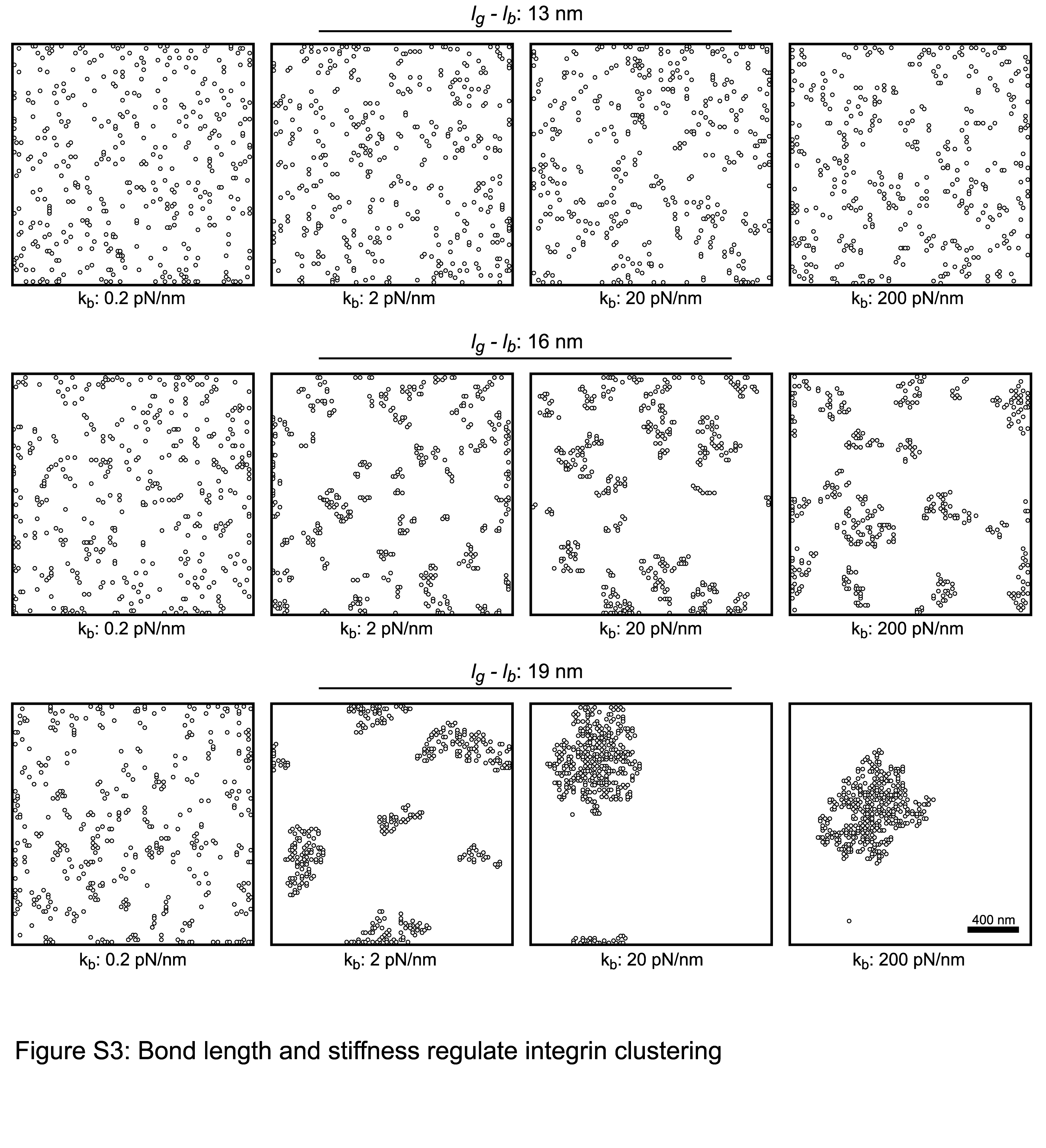

Supplement: Figure S3 — Bond length and stiffness regulate integrin clustering. Steady-state integrin positions acquired by simulating integrin dynamics on rigid substrates with various combinations of integrin bond length and stiffness. See Table 1 for additional parameters. Simulated area: 2 µm×2 µm. (1.25 MB TIF) [file pcbi.1000604.s003.tif]

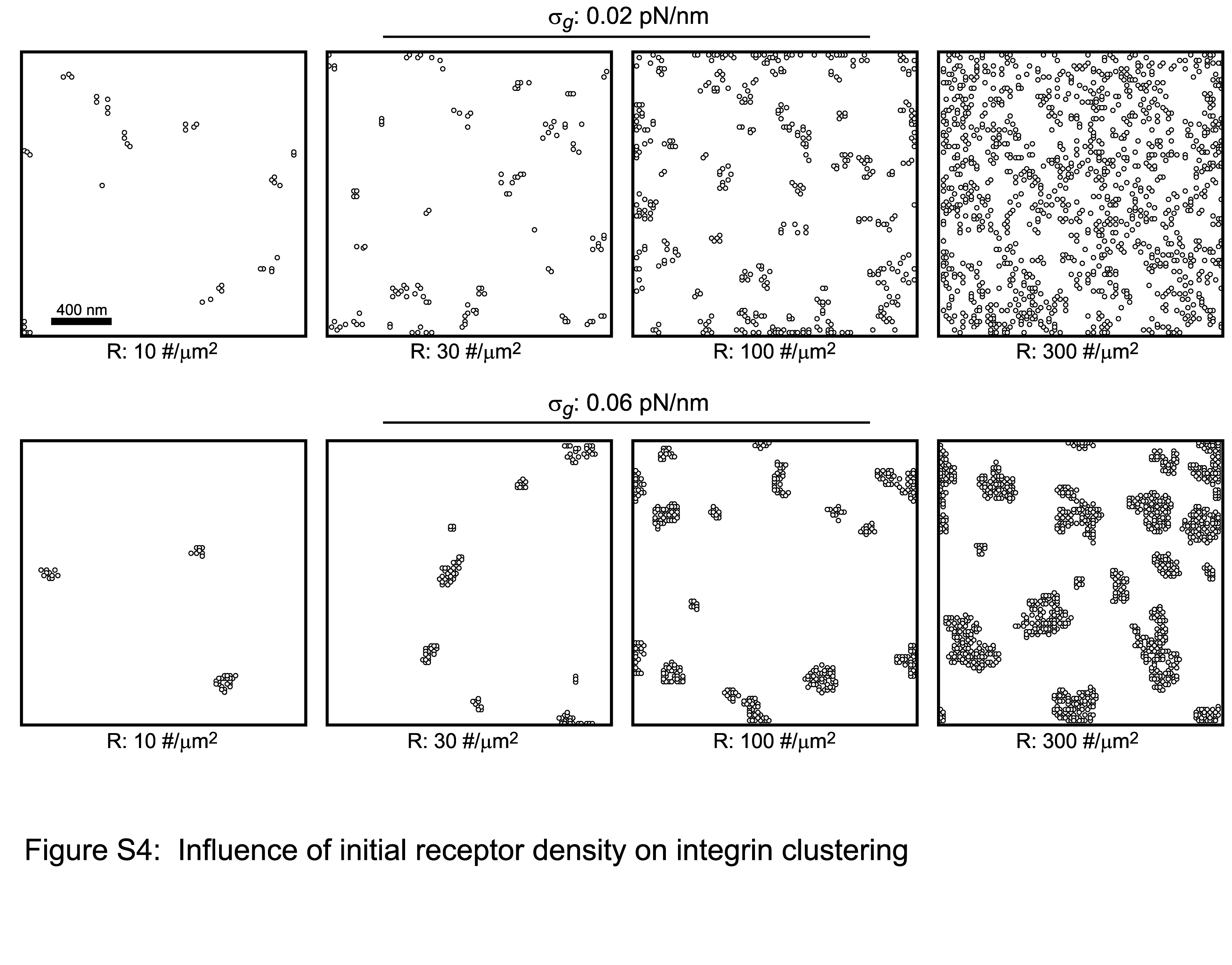

Supplement: Figure S4 — Influence of initial receptor density on integrin clustering. Steady-state integrin positions determined by simulating integrin dynamics on rigid substrates with various initial densities of integrin receptor. All other parameters are best-estimate and listed in Table 1. Simulated area: 2 µm×2 µm. (0.83 MB TIF) [file pcbi.1000604.s004.tif]
